# Supplementary material for: Spatial Gene Expression of Human Coronary Arteries Revealed the Molecular Features of Diffuse Intimal Thickening in Explanted Hearts
Source: Int J Mol Sci. 2025 Feb 24;26(5):1949. doi: 10.3390/ijms26051949 (PMC11900230; doi:10.3390/ijms26051949)
Supplement: Supplementary file 1 [file ijms-26-01949-s001.zip › ijms-3471725-supplementary.pdf]

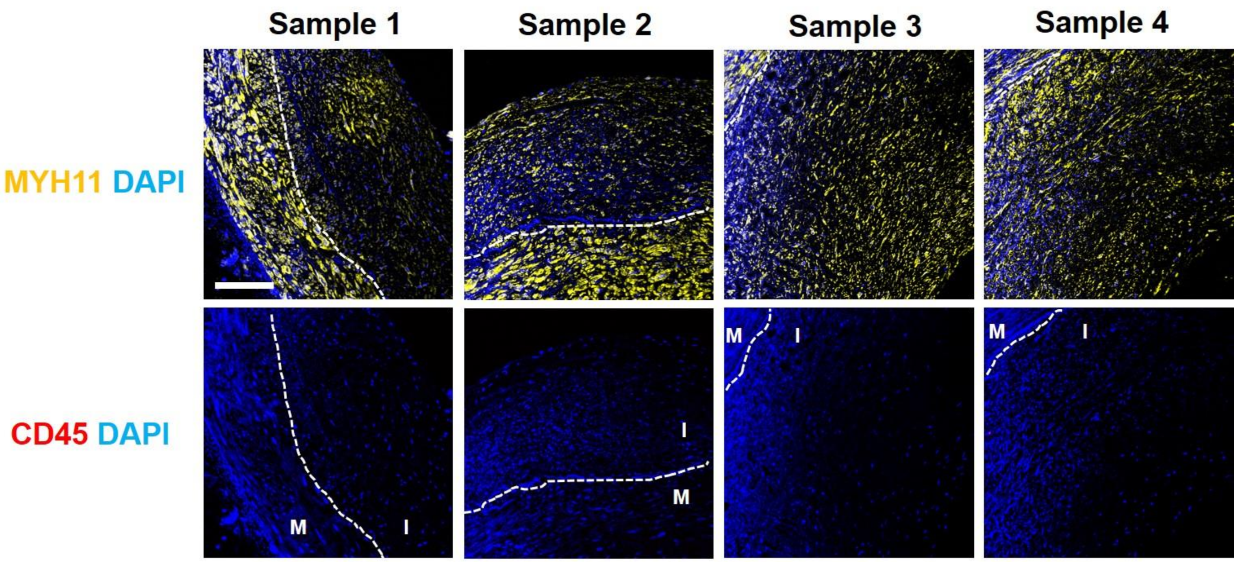

Supplemental Figure S1. Confocal imaging of DIT regions with MYH11 (yellow), CD45 (red), and nuclei (blue). Scale bars = 100 µm. White dash line: internal elastic lamina; I: intima; M: media.

Supplemental Table S1: Characteristics of sample donors.

	Sam ple	Age at donation	Sex	FFPE block storage time (Year)	Pathology diagnosis of heart tissues	Histology of coronary arteries
Autopsy	A1	24	F	2	No report	DIT
Autopsy	A2	69	M	1	Normal	DIT
Autopsy	A3	55	M	0.2	No report	DIT
Autopsy	A4	42	F	2	Normal	PIT
Autopsy	A5	85	M	1	No report	PIT
Autopsy	A6	22	M	0.5	No report	PIT
Autopsy	A7	65	F	1	Hypertrophic cardiomyopathy	PIT
Explanted heart	E1	48	M	1	Ischemic heart disease	DIT
Explanted heart	E2	60	F	3	Idiopathic dialated cardiomyopathy	DIT
Explanted heart	E3	69	M	10	Ischemic heart disease	DIT
Explanted heart	E4	66	F	9	Cardiac sarcoidosis	DIT
Explanted heart	E5	66	M	4	Idiopathic dialated cardiomyopathy	PIT
Explanted heart	E6	45	F	5	Ischemic heart disease	PIT
Explanted heart	E7	64	F	19	Idiopathic dialated cardiomyopathy	DIT

DIT: diffuse intimal thickening; PIT: pathologic intimal thickening

Supplemental Table S2: Top 20 GO Cellular Component for DEGs enriched in coronary arteries from explanted heart vs. autopsy

Term	P-value	Odds Ratio	Combined Score	Genes
Focal Adhesion (GO:0005925)	9.79E-31	10.29373	711.2816	*SCARB2;ITGB1;NCKAP1;CYFIP1;CD81;TGFB1I1;FHL1;ACTB;ACTG1;PPP1CB;GJA1;CSRP1;CDH2;ADGRE5;JAK2;B2M;ANXA1;PPP1R12A;DST;ANXA5;ACTN4;HSPG2;YWHAZ;RHOA;ENAH;PALLD;ITGA8;CDH13;MYH9;TLN1;MAPRE2;CD44;VCL;AHNAK;SYNPO2;NEDD9;NEXN;EGFR;ADD1;RRAS;FLNA;CD99;CLASP2;HSPA8;CAV2;CAV1;MSN;ACTC1;CD9;CSPG4;VIM*
Cell-Substrate Junction (GO:0030055)	2.67E-30	10.05018	684.3814	*SCARB2;ITGB1;NCKAP1;CYFIP1;CD81;TGFB1I1;FHL1;ACTB;ACTG1;PPP1CB;GJA1;CSRP1;CDH2;ADGRE5;JAK2;B2M;ANXA1;PPP1R12A;DST;ANXA5;ACTN4;HSPG2;YWHAZ;RHOA;ENAH;PALLD;ITGA8;CDH13;MYH9;TLN1;MAPRE2;CD44;VCL;AHNAK;SYNPO2;NEDD9;NEXN;EGFR;ADD1;RRAS;FLNA;CD99;CLASP2;HSPA8;CAV2;CAV1;MSN;ACTC1;CD9;CSPG4;VIM*
Vesicle (GO:0031982)	2.36E-13	7.446792	216.5015	*DYNC1I2;AHNAK;CD81;COL12A1;ACTB;EGFR;GNAI2;UBB;RAB4A;ANXA1;ANXA2;MSN;TUBB4B;YWHAZ;RHOA;MYO1D;MYL6;MYO1C;GNB1;OGN;CD9;LGR6;CALM2;VCL;PICALM*
Cell-Cell Junction (GO:0005911)	8.28E-12	5.957296	152.0123	*ITIH5;TINAGL1;COL16A1;COL14A1;ELN;TGFB1I1;SERPINE1;COL12A1;HTRA1;LTBP1;LGALS1;CDH2;TIMP3;CCN2;ANXA1;ANXA2;VWF;DST;ANXA5;NPNT;HSPG2;ASPN;MFAP4;OGN;CDH13;S100A4;CSPG4*
Cytoskeleton (GO:0005856)	1.6E-10	3.93782	88.81784	*PTGIS;CAVIN1;PRKAR1A;CAV2;CAV1;KCNMA1;FXYD1;CDH13;JAK2*
Collagen-Containing	2.17E-10	4.878557	108.5556	*ITGB1;RYR2;CAV2;CAV1;FXYD1;SYNC;SLC8A1;VCL;AQP1*

Extracellular Matrix (GO:0062023)				
Actin Cytoskeleton (GO:0015629)	3.32E-10	5.153745	112.4903	*ITGB1;ACTA2;MYO1D;CLIC4;ACTC1;MYO1C;PDGFA;MSN;CD302;CD44;JAM3*
Adherens Junction (GO:0005912)	3.68E-08	6.759778	115.7065	*CYFIP1;HSPA8;HSP90AA1;HSP90AB1;ANXA2;CAB39;VWF;ROCK1;SERPINE1;PDGFA;ACTN4;TUBB4B;ISLR;SELENOP;TIMP3;MAGED2;CCT8;B2M;FRK;KPNB1;VCL*
Secretory Granule Lumen (GO:0034774)	1.01E-07	4.377175	70.52332	*CYFIP1;COL16A1;HSP90AB1;CAB39;COL14A1;COL12A1;PDGFA;PRSS23;LTBP1;EGFR;GLS;LGALS1;CDH2;ALDH1B1;ESD;CCT8;JAK2;B2M;HSPA8;CRTAP;HSP90AA1;IGFBP3;HSPG2;SOD1;EOGT;OGN;CSPG4;YPEL5;KPNB1;VCL;ISCU*
Actin-Based Cell Projection (GO:0098858)	1.59E-07	9.083288	142.1873	*CLIC4;AHNAK;SYNPO2;NEXN;WASL;ACTB;MYLK;ACTG1;CDH2;CFL2;STK38L;FLNA;PLS3;PDLIM5;ANXA1;PPP1R12A;SEPTIN2;DSTN;ACTN4;MYO1D;ACTC1;MYO1C;PALLD;MYADM;MYH9*
Sarcolemma (GO:0042383)	1.87E-07	12.55878	194.5867	*CLIC4;AHNAK;LDB3;ADD1;ACTG1;TMEM47;CYTH3;GJA1;ARVCF;CDH2;FLNA;CCN3;PDLIM5;JAM3;ANXA1;ANXA2;MSN;WTIP;RHOA;CDH11;MYADM;PECAM1;CDH13;ESAM;TLN1;VCL*
Cytoplasmic Vesicle Membrane (GO:0030659)	7.97E-07	3.683976	51.73129	*CRTAP;COL16A1;COL14A1;IGFBP3;COL12A1;PDGFA;PRSS23;LTBP1;LGALS1;CDH2;EOGT;ESD;CASQ2;B2M*
Membrane Raft (GO:0045121)	1.07E-06	5.47237	75.23785	*COL12A1;GNB1;OGN;CD9;TUBB4B;VCL;GNAI2*

Caveola (GO:0005901)	8.96E-06	8.857272	102.9422	*SCARB2;ITGB1;CD74;RAB4A;SORT1;CAV1;ARHGAP1;WASL;ANTXR1;PKD1;RHOA;EGFR;GJA1;UBB;ADGRE5;SNX17;KIF13A;PECAM1;CD9;PAM;ITM2B;CD44*
Plasma Membrane Raft (GO:0044853)	9.56E-06	7.386327	85.37311	*GJA1;AHNAK;CDH2;FXYD1;SLC8A1;VCL;JAM3*
Cell-Cell Contact Zone (GO:0044291)	1.25E-05	10.4383	117.8227	*ANXA1;ANXA2;MSN;LDB3;WTIP;ADD1;CYTH3;ARVCF;CDH2;CDH11;CDH13;ESAM;TLN1;PDLIM5;VCL*
Vacuolar Lumen (GO:0005775)	1.85E-05	4.850858	52.87269	*MFAP4;ELN;MYH9;LTBP1;MYH10*
Platelet Alpha Granule (GO:0031091)	1.87E-05	6.737615	73.35144	*MYO1D;CLIC4;MYO1C;PDGFA;MSN;CD44;JAM3*
Intracellular Membrane-Bounded Organelle (GO:0043231)	2.24E-05	1.626904	17.42021	*PLN;FXYD1*
Nucleus (GO:0005634)	2.32E-05	1.653406	17.64444	*ARHGEF9;CDH2;PDLIM5;SLC8A1;RTN4;ADD1*

Supplemental Table S3: Top 20 GO Pathways (Bioplanet 2019) for DEGs enriched in intimal SMC-enriched capture spots.

Term	P-value	Odds Ratio	Combined Score	Genes
TGF-beta regulation of extracellular matrix	4.60597E-10	3.765136581	80.94478067	*TNFAIP6;ELN;FGL2;PDGFA;LTBP2;AEBP1;THBS2;PTHLH;RGS2;EFEMP1;CDH2;PPP1R13L;PDGFD;S1PR1;PDGFRA;POSTN;TGFB1;VCAM1;HGF;FN1;AKR1C2;INHBA;TM4SF1;SMAD7;COL1A1;PDP1;SFRP4;EHD3;VCAN;LOX;PPA1;ITGA11;NOX4;SCG2;SKAP1*
Syndecan 1 pathway	9.37502E-09	15.11882716	279.4748045	*COL1A1;COL16A1;TGFB1;COL13A1;COL5A1;CCL5;HGF;COL5A2;COL8A1;MET*
Extracellular matrix organization	1.4582E-07	8.089799297	127.3406696	*COL1A1;ADAMTS2;COL16A1;COL13A1;COL5A1;MMP2;MMP3;COL5A2;COL21A1;COL8A1;FURIN;PLOD1*
RAGE pathway	1.48157E-06	9.570963011	128.4653745	*BACE1;TGFB1;VCAM1;ICAM2;FN1;PDGFA;CCL2;TNFRSF11B;MFGE8*
PDGF genes and receptors	1.67879E-06	107.2568306	1426.24066	*PDGFRB;PDGFRA;PDGFD;PDGFA*
Collagen biosynthesis and modifying enzymes	2.58623E-06	8.873079829	114.1549073	*COL1A1;ADAMTS2;COL16A1;COL13A1;COL5A1;COL5A2;COL21A1;COL8A1;PLOD1*
Malaria	4.04475E-06	10.06655531	125.0073917	*TGFB1;VCAM1;LRP1;HGF;PECAM1;CCL2;THBS2;MET*
Glycosaminoglycan metabolism	6.21938E-06	6.044877747	72.46503059	*ACAN;VCAN;CHPF;OMD;DSE;BGN;CHST1;PRELP;AGRN;FMOD;SLC9A1*
Cytokine-cytokine receptor interaction	9.78038E-06	3.763753084	43.41538957	*PDGFRB;PDGFRA;TGFB1;IFNGR1;HGF;PDGFA;TNFRSF11B;INHBA;IL1A;IL7;IFNE;CCL5;XCL2;CCL2;CCL18;MET;CCR10*
Urokinase-type plasminogen activator (uPA) and uPAR-mediated signaling	1.0823E-05	10.79614325	123.4413726	*PDGFRB;TGFB1;LRP1;PDGFD;HGF;MMP3;FN1*
Interleukin-1 regulation of extracellular matrix	1.43637E-05	5.487490736	61.18996144	*IL1A;LOX;TNFAIP6;NOS2;CCL5;MMP3;CCL2;TNFRSF11B;INHBA;PLXDC2;RUNX1*

ECM-receptor interaction	2.49836E-05	6.500277008	68.88533806	*COL1A1;VWF;COL5A1;ITGA10;COL5A2;ITGA11;FN1;THBS2;AGRN*
Beta-1 integrin cell surface interactions	2.86636E-05	7.457420461	78.0037428	*COL1A1;VCAM1;COL5A1;ITGA10;COL5A2;ITGA11;FN1;THBS2*
Focal adhesion	3.12031E-05	3.762501615	39.03592434	*PDGFRB;PDGFRA;VWF;HGF;FN1;PDGFA;THBS2;COL1A1;CCND3;COL5A1;PDGFD;ITGA10;COL5A2;ITGA11;MET*
Keratan sulfate degradation	3.43145E-05	30.63700234	314.9466033	*ACAN;OMD;PRELP;FMOD*
Interleukin-4 regulation of apoptosis	4.08698E-05	3.489589664	35.2627186	*POSTN;VCAM1;MMP3;FGL2;MX1;FN1;INHBA;SULF1;ACAN;RGS2;VCAN;LOX;S1PR1;CCL2;UPP1;MET*
Integrins in angiogenesis	6.61538E-05	6.550811987	63.04192556	*COL1A1;COL16A1;COL13A1;COL5A1;COL5A2;FN1;COL8A1;MFGE8*
Oncostatin M	7.45123E-05	3.167331522	30.10404802	*NOS2;SLC10A1;ANXA3;HGF;MMP2;IGFBP2;MMP3;FN1;KRT7;PDGFA;GRIK2;PTHLH;COL1A1;CCL5;CDH11;CCL2;LGALS7*
Signaling by PDGF	9.13237E-05	4.84077381	45.02452133	*PDGFRB;COL1A1;PDGFRA;PRKAR1B;COL5A1;PDGFD;COL5A2;PDGFA;FURIN;THBS2*
Adhesion and diapedesis of lymphocytes	9.96033E-05	21.44262295	197.5790828	*IL1A;VCAM1;ICAM2;PECAM1*

Supplemental Table S4: Top 20 GO Pathways (Bioplanet 2019) for DEGs enriched in medial SMC-enriched capture spots.

Term	P-value	Odds Ratio	Combined Score	Genes
Muscle contraction	5.01771E-10	25.87369186	554.0301658	*DES;ACTN2;TPM2;TPM1;LMOD1;MYH11;MYL9;ACTG2;MYLK*
Smooth muscle contraction	6.72925E-10	53.11417625	1121.738863	*TPM2;TPM1;LMOD1;MYH11;MYL9;ACTG2;MYLK*
Striated muscle contraction	1.49414E-09	30.50327553	619.8788388	*MYOM1;ACTA1;ACTC1;DES;ACTN2;TPM2;TPM1;MYL9*
FSH regulation of apoptosis	1.80194E-08	7.130902643	127.1569541	*IGFBP5;IGFBP4;TPM2;TPM1;ACTG2;HSD11B1;AKAP12;ACTA1;PLCE1;TIMP3;FLNC;PTGDS;FILIP1L;CDK14;ANGPTL1*
Vascular smooth muscle contraction	1.06549E-05	8.439734532	96.63069446	*EDNRA;PPP1R12A;MYH11;AVPR1A;PPP1R12B;MYL9;ACTG2;MYLK*
Dilated cardiomyopathy	3.48926E-05	8.533061426	87.5768209	*ACTC1;DES;TPM2;TPM1;PRKAG2;ITGA7;SLC8A1*
Focal adhesion	5.40005E-05	5.138856109	50.49705706	*RAP1A;PPP1R12A;TNXB;ACTN2;TNC;ITGA7;FLNB;FLNC;MYL9;MYLK*
Myometrial relaxation and contraction pathways	8.55457E-05	6.188352798	57.96295778	*CNN1;ACTA1;RGS19;ACTC1;IGFBP5;IGFBP4;PDE4D;SLC8A1*
Alternative complement pathway	0.000114027	41.7366573	378.9303555	*C3;CFD;C7*
cAMP cell motility pathway inferred from amoeba model	0.000352331	9.052877566	71.97888824	*ACTA1;ACTC1;PDE4D;PLCE1;ACTG2*
Integrin signaling pathway	0.000534567	5.347041007	40.28489633	*ACTA1;RAP1A;ACTC1;ACTN2;PPP1R12B;ACTG2;MYLK*

Gastrin-CREB signaling pathway via PKC and MAPK	0.000590708	4.58247212	34.06696447	*DGKG;NPSR1;EDNRA;DGKD;RGS19;GPR68;NMUR1;AVPR1A*
Beta-oxidation of lauroyl-CoA to decanoyl-CoA	0.000800036	73.80260708	526.275571	*HADHB;HADH*
Creatine metabolism	0.001660143	44.27709497	283.4111213	*GATM;CKB*
Fatty acid elongation in mitochondria	0.002200366	36.89571695	225.769753	*HADHB;HADH*
Mitochondrial beta-oxidation of saturated fatty acids	0.002200366	36.89571695	225.769753	*HADHB;HADH*
BDNF signaling pathway	0.002659117	3.576229752	21.20618795	*TAGLN;IGFBP5;C7;IGFBP4;RORB;FOXN3;TGFBI;FLNC*
Prostaglandin biosynthesis and regulation	0.002719597	11.91272071	70.37167719	*HSD11B1;EDNRA;PTGDS*
Seven transmembrane receptor signaling through beta-arrestin	0.004561592	6.375786925	34.36602439	*ACTA1;ACTC1;PDE4D;ACTG2*
G alpha s pathway	0.004736602	4.867588933	26.05345494	*PYGB;RAP1A;RGS19;PDE4D;CNGA3*

Supplemental Table S5: Overlapped genes between DEGs of SMC-enriched capture spots in DIT and signature genes of SMC sub-clusters from atherosclerotic lesions.

SMC-enriched capture spots in the DIT	Compared to top 100 specific markers	Numbers of overlapped genes	Overlapped genes
Intima	$Ldlr^{-/-}Apob^{100/100}$ mice $Sca1^{+}$ SMC	19	*VCAM1, TNFRSF11B, COL8A1, EFEMP1, MMP3, LTBP2, MMP2, SFRP4, FN1, FXYD5, SCARA3, MXRA8, LOX, ALCAM, COL5A2, COL1A1, THBD, PLXDC2, FUCA1*
	$Apoe^{-/-}$ mice $Sca1^{+}$ SMC	12	*BGN, HGF, COL1A1, VCAM1, TM4SF1, MGP, BRINP1, CDH11, RUNX1, EFEMP1, FAP, COL5A2*
	Human carotid plaques sub-cluster 1	3	*SUSD5, MRAP2,SCARA3*
	Human carotid plaques sub-cluster 2	4	*SUSD5, TMEM130, TPH1, MRAP2*
	Human carotid plaques sub-cluster 3	15	*HMCN1, FGL2, OMD, LTBP4, ITGA10, ELN, PDP1, FMOD, MRC2, LRP1, AKR1C2, S1PR1, CALCRL, SUGCT, SPARC*
	Human carotid plaques sub-cluster 4	14	*ARHGDIB, TM4SF1, VCAN, HTRA1, MRAP2, MFGE8, LOX, HAPLN3, FKBP10, FMOD, PLOD1, CHPF, FAP, COL5A2*
	Human carotid plaques sub-cluster 5	37	*IGFBP3, ELN, MASP1, MEGF6, COL8A1, INHBA, ART4, KRT7, ENG, LTC4S, VCAN, PROCR, LRRC32, COL1A1, SPARC, SLC14A1, CHPF, TNFRSF11B, PRSS23,*

			COL5A1, GALNT2, EFEMP1, ITGA10, ADAMTS2, EHD3, CDH2, FKBP10, FN1, FGF1, DAP, PLOD1, CCN2, POSTN, TMEM204, ITGA11, SULF1, FOXS1
	Human carotid plaques sub-cluster 6	17	*SFRP4, OMD, LTBP2, MMP2, FGL2, SCG2, MXRA5, TPPP3, MARCKS, MXRA8, COL5A2, PDGFD, TNFAIP6, MRC2, COL1A1, LRP1, AKR1C2*
	Human carotid plaques sub-cluster 7	19	*POSTN, MXRA5, COL1A1, COL5A1, CTHRC1, LOXL2, FAP, HEG1, SULF1, FN1, COL5A2, PLOD1, VCAM1, SFRP4, MMP2, PRSS23, RGS2, LOX, CDH11*
	Human carotid plaques sub-cluster 8	5	*CCL2, CALCRL, TPPP3, IL34, PHLDA1*
Media	$Ldlr^{-/-}Apob^{100/100}$ mice $Sca1^{+}$ SMC	4	*C3, SNCG, TNC, IGFBP4*
	$Apoe^{-/-}$ mice $Sca1^{+}$ SMC	1	*C3*
	Human carotid plaques sub-cluster 1	26	*ACTC1, CNN1, C11orf96, MYH11, MCAM, ACTG2, PPP1R12B, FILIP1L, SBSPON, SMTN, SORBS2, LMOD1, MYLK, MYL9, SYNPO2, ITGA7, LPP, C12orf75, LDB3, DSTN, TPM2, PPP1R12A, HSPB6, PHGDH, FILIP1, SLMAP*

	Human carotid plaques sub-cluster 2	19	*MYH11, C12orf75, CNN1, LMOD1, PPP1R12B, MCAM, SORBS2, MYL9, FILIP1L, TPM2, LPP, DSTN, MYLK, SYNPO2, SLMAP, TAGLN, HSPB6, ACTG2, PPP1R12A*
	Human carotid plaques sub-cluster 3	2	*IGFBP5, BMPR1B*
	Human carotid plaques sub-cluster 4	2	*RARRES2, SMTN*
	Human carotid plaques sub-cluster 5	1	*AKAP12*
	Human carotid plaques sub-cluster 6	7	*CCDC80, FBLN1, SERPINF1, PTGDS, TNXB, TIMP3, TNC*
	Human carotid plaques sub-cluster 7	7	*TIMP3, TNC, SERPINF1, PTGDS, TGFBI, IGFBP4, CCDC80*
	Human carotid plaques sub-cluster 8	9	*C7, C3, TGFBI, IGFBP5, TNC, IGFBP4, ABCC9, SERPINF1, VPS16*

Supplemental Table S6: Unique top 20 signature genes of Sca1$^+$ SMC not overlapped with DEGs of intimal SMC-enriched capture spots.

	**Non overlapped genes**
Ldlr$^{-/-}$Apob$^{100/100}$ mice Sca1$^+$ SMC	*LUM,C3,SERPINA3N,DCN,SPP1,LCN2,C4B,CP,CXCL12,TIMP1, FRZB,LY6A,THBS1,TMEM176B,CX3CL1, ICAM1*
Apoe$^{-/-}$ mice Sca1$^+$ SMC	*C3,SERPINA3N,LUM,GPR88,CAR13,LCN2,SPP1,PLSCR1,ART4,PTK2B,CCL9,GPX3,H2-DMA,LY6A,CST6,RAI14,STING1,SDSL,LY6C1,H2-EB1*
